# Vascular and nerve biomarkers in thigh skin biopsies differentiate painful from painless diabetic peripheral neuropathy

**DOI:** 10.3389/fpain.2024.1485420

**Published:** 2024-10-25

**Authors:** Gordon Sloan, Philippe Donatien, Rosario Privitera, Pallai Shillo, Sharon Caunt, Dinesh Selvarajah, Praveen Anand, Solomon Tesfaye

**Affiliations:** ^1^ Division of Clinical Medicine, University of Sheffield, Sheffield, United Kingdom; ^2^ Diabetes Research Unit, Sheffield Teaching Hospitals NHS Foundation Trust, Sheffield, United Kingdom; ^3^ Peripheral Neuropathy Unit, Imperial College London, Hammersmith Hospital, London, United Kingdom

**Keywords:** pain, biomarkers, skin, vascular, painful diabetic neuropathy, von Willebrand factor, IENF, type 2 diabetes

## Abstract

**Background:**

Identifying distinct mechanisms and biomarkers for painful diabetic peripheral neuropathy (DPN) is required for advancing the treatment of this major global unmet clinical need. We previously provided evidence in calf skin biopsies that disproportion between reduced sensory small nerve fibers and increased blood vessels may distinguish painful from non-painful DPN. We proposed that overexposure of the reduced nerve fibers in DPN to increased hypoxemia-induced vasculature and related algogenic factors, e.g., nerve growth factor (NGF), leads to neuropathic pain. To further investigate this proposed mechanism, we have now studied more proximal thigh skin biopsies, to see if the same disproportion between increased vasculature and decreased nerve fibers generally differentiates painful DPN from painless DPN.

**Methods:**

A total of 28 subjects with type 2 diabetes (T2DM) and 13 healthy volunteers (HV) underwent detailed clinical and neurophysiological assessments, based on the neuropathy composite score of the lower limbs [NIS(LL)] plus 7 tests. T2DM subjects were subsequently divided into three groups: painful DPN (*n* = 15), painless DPN (*n* = 7), and no DPN (*n* = 6). All subjects underwent skin punch biopsy from the upper lateral thigh 20 cm below the anterior iliac spine.

**Results:**

Skin biopsies showed decreased PGP 9.5-positive intraepidermal nerve fiber (IENF) density in both painful DPN (*p* < 0.0001) and painless DPN (*p* = 0.001). Vascular marker von Willebrand Factor (vWF) density was markedly increased in painful DPN vs. other groups, including painless DPN (*p* = 0.01). There was a resulting significant decrease in the ratio of intraepidermal nerve fiber density to vasculature and PGP9.5 to vWF, in painful DPN vs. painless DPN (*p* = 0.05). These results were similar in pattern to those observed in these HV and T2DM groups previously in distal calf biopsies; however, the increase in vWF was much higher and nerve fiber density much lower in the calf than thigh for painful DPN. Thigh skin vWF density was significantly correlated with several metabolic (waist/hip ratio, HbA1c), clinical (e.g., pain score), and neurophysiological measures.

**Conclusion:**

This study supports our proposal that increased dermal vasculature, and its disproportionate ratio to reduced nociceptors, may help differentiate painful DPN from painless DPN. This disproportion is greater in the distal calf than the proximal thigh skin; hence, neuropathic pain in DPN is length-dependent and first localized to the distal lower limbs, mainly feet.

## Introduction

Diabetic peripheral neuropathy (DPN) is the most common chronic complication of diabetes (1). Up to half of people with DPN develop neuropathic pain (painful DPN), which causes a variety of unpleasant symptoms in the legs and feet, including constant burning pain, paroxysmal “electric-shock”-like pain, and hypersensitivity to the touch of normally benign stimuli (allodynia) (2, 3). These symptoms are often more intense at night and thus can induce sleep impairment (4, 5). Unsurprisingly, these symptoms can lead to mood impairment and a reduction in quality of life (5, 6). In addition to the profound impact of painful DPN on patients, this condition is also associated with greater healthcare costs than with people who have diabetes or DPN alone (7).

Our understanding of DPN remains incomplete and treatment is limited (1, 8). Recent publication reports of topical high-dose capsaicin treatments show pain relief and disease modification (9–15), although there are no licensed disease-modifying therapies for DPN (16). The current recommended treatments all aim to improve neuropathic pain symptoms; however, they have limited efficacy, which is offset by dose-related adverse events (17). Thus, there is a sound rationale for investigating the underlying mechanisms of painful DPN and identifying new targets for therapeutic interventions.

Recent studies have included immunohistochemical analysis of skin biopsy samples as a method to investigate the pathophysiology of painful DPN (18–24). Intraepidermal nerve fibers (IENF) are now readily identified on skin biopsy samples using the pan-neuronal marker protein gene product 9.5 (PGP 9.5) (25). Skin biopsy with PGP 9.5 analysis is a recommended investigative technique for the diagnosis of small fiber neuropathy, including in diabetes (26, 27). In DPN, there is a reduction in IENF density (28), but IENF and other measures of neurological function, e.g., quantitative sensory testing (QST), are unhelpful in distinguishing between painful and painless DPN (20, 22, 29–31). However, we and other groups have demonstrated additional markers in skin biopsies that may differentiate between painful and painless DPN (31) such as markers of nerve degeneration (axonal swellings) and nerve regeneration (GAP-43) (20, 21, 32). An increase in PGP 9.5 positive nerve fibers towards normal density correlated positively with pain relief in patients treated with high-dose capsaicin patches (10, 11).

We recently demonstrated that subjects with painful DPN had a significantly higher blood vessel density, measured using von Willebrand factor (vWF), compared with painless DPN (*p* < 0.001), whereas there was no group difference in other neuronal markers (e.g., IENF density or GAP-43) (24). This novel finding suggests that hypoxemia-induced increase in blood vessels may be responsible for an excessive exposure of depleted small nerve fiber terminals to key algogenic substances related to or derived from increased blood vessels, e.g., NGF, in painful DPN. The calf skin biopsies in our previous study were collected 10 cm above the ankle [as per the European Federation of Neurological Societies/Peripheral Nerve Society Guidelines (33)] in subjects with advanced diabetes, in whom IENF was markedly reduced. Therefore, we have now performed skin biopsies at the proximal thigh, where IENF would be less reduced, to investigate the density of vascular and neural biomarkers in HV, painful-DPN, painless-DPN, and no-DPN subgroups and a general relationship to neuropathic pain.

## Materials and methods

### Study design and participants

Forty-one participants were recruited to the study, including 13 healthy volunteers (HV) and 28 with type 2 diabetes mellitus T2DM [15 painful DPN, 7 painless DPN, and 6 T2DM without DPN (no DPN)]. Patients were recruited from Sheffield Teaching Hospitals NHS Foundation Trust Diabetes Databases and outpatient clinics, and healthy volunteers were recruited by self-referral from promotional materials. All participants with T2DM were diagnosed according to the World Health Organization criteria for at least 6 months prior to their inclusion into the study. The exclusion criteria included as follows: current or past history of excessive alcohol usage (>14 units/week), non-diabetic neuropathies, other neurological disorders that may confound clinical assessments, pregnancy, major lower limb amputation, eGFR <45 ml/min/1.73 m^2^, absence of peripheral pulses, use of anticoagulation medications, known bleeding disorder, and moderate-to-severe pain from causes other than DPN. All participants gave written, informed consent before participation in the study, which had prior ethics approval by the Nottingham Research Ethics Committee (reference no. 17/EM/0430).

Participants underwent detailed clinical and neurophysiological assessments, including the following: (1) Toronto Clinical Neuropathy Score (TCNS) to assess neuropathy symptoms and signs (34); (2) Neuropathy Impairment Score Lower Limb [NIS(LL)] to assess clinical peripheral neurological status (35); (3) Douleur Neuropathique en 4 (DN4) questionnaire to assess neuropathic pain symptoms and signs (36); (4) Neuropathic Pain Symptom Inventory (NPSI) to evaluate different symptoms of neuropathic pain (37); (5) nerve conduction studies (NCS) performed at a stable skin temperature of 31°C and a room temperature of 24°C, using an electrophysiological system [Medelec; Synergy Oxford Instruments, Oxford, UK. The following peripheral nerve attributes were measured: (a) sural sensory nerve action potential; (b) common peroneal motor nerve distal latency, compound muscle action potential, and conduction velocity; and (c) tibial motor nerve distal latency]; (6) sudomotor assessment with measurement of foot electrochemical skin conductance (ESC) using SUDOSCAN (Impeto Medical, Paris, France), as a marker of peripheral autonomic small fiber neuropathy (38); (7) vibration detection thresholds acquired from the dorsal aspect of the right foot using the Computer-Assisted Sensory Evaluation IV (WR Electronics, Stillwater, MN, USA) using the four-, two-, and one-step algorithm with null stimuli, based on comparative data from computer simulation and patient responses (39); (8) cardiac autonomic function tests performed using a computer-assisted technique (40); and (9) German Research Network of Neuropathic Pain (DFNS) quantitative sensory testing (41).

The DFNS QST was performed as per the protocol (41), briefly described as follows (42). Cold (CDT) and warm detection thresholds (CDT), as well as cold (CPT) and heat pain thresholds (CPT) and thermal sensory limens (TSL) and paradoxical heat sensations (PHS), were established using a MEDOC TSA-II Neurosensory Analyzer (Ramat Yishai, Israel). The thermode probe area contacting the skin was 30 mm × 30 mm, and the range of stimulus intensity ranged from 0°C to 50°C. We also tested mechanical detection (MDT), pain thresholds (MPT), mechanical pain sensitivity (MPS), dynamic mechanical allodynia (DMA), pressure pain thresholds (PPTs), wind-up ratio (WUR), and vibration detection thresholds (VDT). The mechanical detection threshold was assessed with a set of standardized von Frey filaments (0.25, 0.5, 1, 2, 4, 8, 16, 32, 64, 128, and 256 mN; Nervtest, Marstock, Germany) using a modified method of limits. The mechanical pain threshold was assessed with a set of seven metal probes of standardized stimulus intensities (8, 16, 32, 64, 128, 256, and 512 mN; MRC Systems, Medizintechnische Systeme, Heidelberg, Germany), using a uniform skin contact area of 0.25 mm and a modified method of limits. The mechanical pain sensitivity of the skin and dynamic mechanical allodynia were determined using the same set of seven metal probes with standardized stimulus intensities and, in addition, a set of three light intensity stimuli: a cotton wool ball with a force of 3 mN; a Q-tip (fixed to a plastic stick) with a force of 100 mN; and a paintbrush with an applied force of 200–400 mN. These stimuli were applied 50 times with a ∼10 s interstimulus interval (five runs of 10 stimuli per test site in different pseudorandomized sequences), and the participants were asked to rate the intensity of each stimulus on a 0–100 numeric rating scale (0, no pain; 100, most severe pain). The WUR, as a measure of enhanced temporal summation, was examined by a pinprick stimulus of standardized intensity (256 mN). The stimulus was first applied singularly and then in a series of 10 stimuli with a frequency of 1 Hz within an area of 1 cm^2^. Participants were asked to rate the intensity of the first stimulus and the mean of ten stimuli on a scale of 0–100. The ratio between the two measures was calculated as WUR; a WUR of >1 indicates enhanced temporal summation. The vibration detection threshold was examined using a tuning fork (64 Hz, 8/8 scale) at the (lateral or medial) malleolus area. The muscular pressure pain threshold was examined by applying mechanical pressure at a rate of 0.5 kg/s (Algometer, Somedic, Sweden) at the abductor hallucis muscle. As per the DFNS protocol, all modalities were tested using the same technique at the dorsum of the foot, except for vibration detection thresholds in which the tuning fork is placed on the medial malleolus and pressure pain threshold where the pressure algometer is placed on the abductor hallucis muscle. The QST data were entered into the data analysis system eQUISTA provided by the DFNS (43). eQUISTA transformed the raw QST data into *z*-scores, thus normalizing for age, sex, and the body location of testing. Positive *z*-scores denote the gain of function, whereas negative *z*-scores denote the loss of function. Formal training for the protocol was obtained at Bochum Hospital, Germany.

Sensory tests (clinical/neurophysiological/QST) were performed in the feet and lower legs rather than the thigh, because the tests were validated based on this region.

Furthermore, the reason for doing the thigh biopsy was to determine potential early length-dependent skin neuronal/vascular changes, in relation to advanced neuropathic processes observed distally with more severe nerve fiber loss.

A neuropathy composite score known as the NIS(LL) + 7 was calculated which combines the NIS-LL with seven neurophysiological tests [cardiac autonomic function, NCS measures, and vibration detection thresholds (VDT)] to determine an overall measure of neuropathy severity (44). The full details of this procedure are detailed elsewhere (35, 44). The NIS(LL) + 7 is a validated, continuous measure of neuropathy severity that has been widely used in DPN research studies as a clinical endpoint.

Based on these assessments, the participants with diabetes were divided into three groups (26):

(1) Confirmed painless DPN, consisting of participants with painless DPN, with abnormal clinical findings (TCNS > 5), and at least two abnormalities on nerve conduction studies one of which had to be an attribute of the sural nerve.
(2) Confirmed painful DPN, with abnormal clinical findings (TCNS > 5) and at least two abnormalities on nerve conduction studies one of which had to be an attribute of the sural nerve, DN4 score ≥4, and chronic neuropathic pain for at least 6 months (45).
(3) No DPN, consisting of participants with no evidence of DPN on clinical scoring or nerve conduction studies, with no evidence of chronic neuropathic pain.

### Thigh skin biopsy and immunohistochemistry

Skin biopsy samples were obtained from all participants in line with the guidelines published by the European Federation of Neurological Societies (EFNS) on the use of skin biopsy in the diagnosis of peripheral neuropathies (33). The skin was anesthetized by local infiltration of 2% lidocaine before a 3 mm punch biopsy was obtained from the upper lateral aspect of the thigh 20 cm below the anterior iliac spine.

The skin biopsy specimen was fixed for 12–18 h in Zamboni fixative and cryprotected overnight (15% sucrose in 0.1 M phosphate buffer) at 4°C and then snap frozen in liquid nitrogen in optimum cutting tissue embedding medium (Tissue-Tek OCT, RA Lamb Ltd., Eastbourne, UK). Frozen sections of 15 µm thickness for vWF antibody (rabbit, 1:10,000; Novocastra Laboratories, Milton Keynes, UK) were collected onto poly-L-lysine (Sigma, Poole, UK)-coated glass slides and post-fixed in freshly prepared, 4% w/v paraformaldehyde in 0.15 M phosphate-buffered saline (PBS) for 30 min. Endogenous peroxidase was blocked by incubation in methanol containing 0.3% w/v hydrogen peroxide for 30 min. After rehydration, appropriately processed sections were incubated overnight with primary antibodies. For PGP9.5 (rabbit, RA95/06, 1:40,000; Ultraclone, Isle of Wight, UK), 50 µm sections were floated onto 4% w/v paraformaldehyde in 0.15 M phosphate PBS for 1 h 30 min in 12-well plates, washed with PBS, and incubated with PGP 9.5 antibodies overnight. They were then washed and incubated with a second antibody for 1 h 30 min and washed again. In all sections, adherent or free floating, sites of primary antibody attachment were revealed using nickel-enhanced, avidin–biotin peroxidase (Vector Laboratories, Peterborough, UK) (46, 47). In PGP 9.5-stained 50 µm sections, the IENF quantification method used followed the EFNS guidelines (33).

Intraepidermal nerve fibers were counted along the length of four non-consecutive sections. The length of epithelium in each counted section was measured using computerized microscopy software (Olympus ANALYSIS 5 Soft, Olympus UK, Southend, Essex, UK), and the results were expressed as fibers/mm length of the section.

The gray-shade detection threshold was applied for quantitation of immunostaining for the vascular marker vWF and was set at a constant level to allow the detection of positive immunostaining. The area of highlighted immunoreactivity was obtained as a percentage (% area) of the field scanned. Three random sections of good architectural tissue preservation (as objectivated by neutral red counterstain), sufficient length (around 3 mm), and good overall staining including no excess background were selected. Images were captured (×40 objective magnification) along the entire length of the sections, and the mean values were used for statistical analysis. Quantification was performed by two independent blinded observers, and there was no significant difference between observers.

### Statistical analysis

The statistical package SPSS version 28 (SPSS, IBM Corp., NY, USA) was used for baseline data. Baseline participant characteristics were described as mean and standard deviation (±) for normally distributed variables, median and interquartile range for variables with a non-parametric distribution, and percentages for categorical variables. We used Spearman's rank correlation for non-parametric variables. One-way ANOVA was used to compare characteristics for parametric data and the Kruskal–Wallis test for non-parametric data. The *χ*^2^ test was used for the analysis of categorical data.

Immunocytochemistry data were analyzed using GraphPad Prism version 5.0 for Windows (GraphPad Prism Software, San Diego, CA, USA). The statistical tests used were paired two-tailed Mann–Whitney test, Student's *t*-test, and two-way ANOVA analysis. For all statistical tests, *p*-values of ≤0.05 were considered significant.

## Results

Table 1 demonstrates the demographic, clinical, and biochemical results from the participants who underwent the study. There was a greater proportion of participants with retinopathy in the no-DPN (*χ*^2^ test, *p* = 0.019) and painful-DPN groups (*p* < 0.001) compared with the painless-DPN group. Moreover, there was a greater proportion of patients with nephropathy in the painless-DPN group compared with the no-DPN group (*p* = 0.027), and the urinary ACR was greater in all diabetes groups compared with the HV group, as expected. The waist/hip ratio and HbA1c were also statistically higher in all the diabetes groups compared with the HV group (all; LSD, *p* < 0.05). Finally, the cholesterol level was statistically lower in the painless-DPN (*p* = 0.001) and painful-DPN groups (*p* = 0.007) compared with the HV group.

**Table 1:** Clinical details of participants.

	HV (*n* = 13)	No DPN (*n* = 7)	Painless DPN (*n* = 6)	Painful DPN (*n* = 15)	*p*-value
Age (years)	63.0 ± 12.3	64.4 ± 4.7	63.1 ± 5.9	65.0 ± 10.2	0.944 A
Sex (% female)	53.8	40.0	25.0	47.1	0.620 *χ*^2^
Duration DM (years)		9.8 ± 5.8	16.3 ± 7.7	11.2 ± 6.8	0.177 A
Retinopathy presence (% present)		80%	62.5%	76.5%	**<0**.001 *χ*^2^*^,†^
Retinopathy score (0 = No DR, 1 = Bck/Pre-P, 2 = Pro/Laser)		0 = 20% 1 = 80% 2 = 0%	0 = 37.5% 1 = 37.5% 2 = 25.0%	0 = 23.5% 1 = 47.1% 2 = 29.4%	**<0**.001 *χ*^2^*^,†^
Nephropathy presence (% present)		20%	50%	47.1%	**<0**.001 *χ*^2^*
ACR (mg/mmol)	0.0 (0.2)	1.5 (1.7)	0.9 (1.9)	1.4 (4.0)	**<0**.**001 KW**
Number of hypoglycaemic episodes in the last 12 months		0 ± 0	3.7 ± 7.5	4.7 ± 12.8	0.791 A
Smoked ever (% yes)	38.5%	40%	75%	76.5%`	0.110 *χ*^2^
Pack-years smoking [Packs (1 pack = 20 cigarettes) × Number of years]	14.2 ± 9.7	15.3 ± 20.9	31.2 ± 22.2	32.1 ± 23.7	0.298 A
Alcohol intake (units/week)	4.4 ± 5.1	10.7 ± 3.1	6.3 ± 4.8	6.5 ± 5.6	0.337 A
Waist/hip ratio	0.86 ± 0.07	0.95 ± 0.07	1.00 ± 0.08	0.96 ± 0.07	**<0**.**001 A**
Body mass index (kg/m^2^)	27.1 ± 6.1	30.8 ± 4.1	30.1 ± 3.2	30.6 ± 6.6	0.362 A
Systolic blood pressure (mmHg)	132.5 ± 21.0	124.2 ± 10.3	137.4 ± 19.7	136.0 ± 14.2	0.538 A
Creatinine (μmol/L)	71.8 ± 8.1	77.4 ± 8.6	72.8 ± 11.2	77.6 ± 22.3	0.746 A
Total Cholesterol (mmol/L)	5.1 ± 1.0	4.1 ± 1.2	3.5 ± 0.7	4.1 ± 0.9	**0**.**004 A**
HbA1c (mmol/mol)	37.4 ± 4.4	56.6 ± 10.6	63.1 ± 16.8	67.4 ± 18.9	**0**.**001 A**

Boldface denotes *p* < 0.05.

* *p* < 0.05, no DPN vs. painless DPN.

^†^ *p* < 0.05, painful DPN vs. painless DPN.

**Figure 1:**
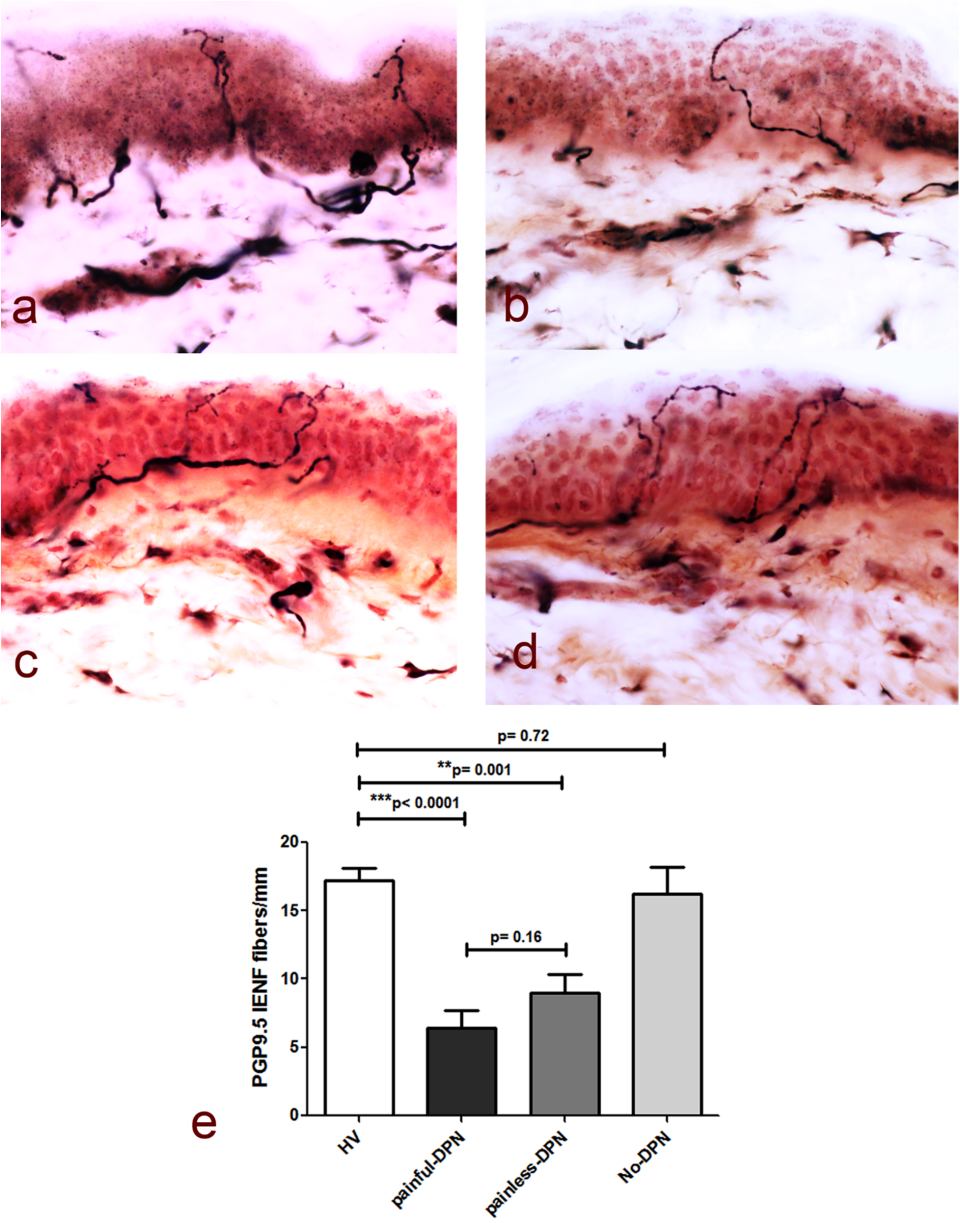
**(a)** PGP9.5 immunoreactivity in thigh skin biopsies from the healthy volunteers and the HV, **(b)** painful-DPN, **(c)** painless-DPN, and **(d)** no-DPN groups. Magnification, ×40. **(e)** Mean ± SEM of the PGP9.5 intraepidermal nerve fibers (fibers/mm).

Table 2 demonstrates the neurological assessments of participants undergoing the study, including the PGP 9.5 immunoreactivity results. As expected, measures of neuropathic pain, i.e., NPSI and DN4, were higher in the painful-DPN group. The TCNS was also statistically higher in the painful-DPN vs. painless-DPN groups. Measures of neuropathy were all statistically higher in the DPN groups compared with non-DPN groups, with no difference between painful- and painless-DPN groups. Figure 1 demonstrates the PGP 9.5 immunoreactivity in the thigh skin from all four study groups. There was a reduction in PGP immunoreactivity in the DPN groups compared with non-neuropathy groups (all; LSD, *p* < 0.001). However, there was no significant difference between the painless- and painful-DPN groups (*p* = 0.208).

**Table 2:** Neurological assessments of study participants undergoing skin biopsy.

	HV (*n* = 13)	No DPN (*n* = 7)	Painless DPN (*n* = 6)	Painful DPN (*n* = 15)	*p*-value
NPSI (total score)	0.0 (0.0)	0.0 (0.0)	0.0 (0.0)	20.8 (18.6)	**<0**.**001 KW**
DN4	0.0 (0.0)	0.0 (0.0)	2.0 (1.5)	6.0 (3.5)	**<0**.**001 KW**
TCNS	0.0 (0.0)	2.0 (1.0)	8.0 (10.0)	15.0 (4.0)	**<0**.**001 KW**
NIS-LL	0.0 (0.0)	0.0 (3.0)	9.0 (19.3)	16.5 (10.0)	**<0**.**001 KW**
NIS-LL + 7	0.0 (1.0)	0.0 (1.5)	17.0 (23.7)	25.2 (13.0)	**<0**.**001 KW**
Peroneal CMAP (mV)	5.1 ± 1.7	5.4 ± 2.7	2.8 ± 3.2	2.1 ± 1.7	**0**.**002 A**
Peroneal MNCV (m/s)	47.3 ± 3.3	46.7 ± 2.9	37.4 ± 9.7	38.9 ± 6.7	**<0**.**001 A**
Peroneal MNDL (msec)	4.9 (0.8)	4.5 (0.9)	6.6 (2.6)	6.1 (4.7)	**0**.**005 KW**
Tibial MNDL (msec)	4.4 (1.2)	4.7 (0.5)	7.4 (2.9)	6.8 (2.6)	**<0**.**001 KW**
Sural SNAP (mV)	16.0 ± 6.4	11.1 ± 3.9	5.6 ± 4.9	2.9 ± 5.7	**<0**.**001 A**
Foot ESC (μS)	75.1 ± 10.9	74.2 ± 13.5	54.1 ± 19.8	43.4 ± 18.9	**<0**.**001 A**
PGP 9.5 IENF fibres/mm	17.4 ± 3.2	17.7 ± 3.6	7.9 ± 4.2	5.4 ± 5.2	**<0**.**001 A**

Boldface denotes *p* < 0.05.

Table 3 demonstrates the DFNS QST parameters of all study participants. As expected, there was evidence of loss of sensory function in the DPN groups compared with the non-neuropathy groups, although MDT was not significantly different from the painless-DPN and other groups. Also, there were no differences in the painless- and painful-DPN groups.

**Table 3:** German pain research network QST results in study participants.

PGP 9.5	HV (*n* = 13)	No DPN (*n* = 7)	Painless DPN (*n* = 6)	Painful DPN (*n* = 15)	*p*-value
CDT *z*-score	−0.82 ± 1.12 (−3.54, 0.61)	−0.78 ± 0.43 (−1.33, −0.26)	−2.21 ± 1.18 (−3.94, −1.02)	−2.13 ± 1.26 (−3.34, 1.40)	**0**.**006 A**
CDT% abnormal	7.7%	0.0%	50%	76.5%	**0**.**001 Chi^2^**
WDT *z*-score	−1.13 ± 0.87 (−2.31, 0.26)	−0.54 ± 0.77 (−1.41, 0.58)	−1.68 ± 1.12 (−2.75, 0.44)	−2.03 ± 0.49 (−2.75, −1.17)	**0**.**002 A**
WDT% abnormal	23.1%	0%	50%	58.8%	0.051 Chi^2^
TSL *z*-score	−1.48 ± 1.09 (−3.72, 0.45)	−1.09 ± 0.27 (−1.34, −0.69)	−2.25 ± 1.30 (−3.94, −0.53)	−2.23 ± 0.59 (−3.18, −1.16)	**0**.**027 A**
TSL% abnormal	30.8%	0%	37.5%	64.7%	**0**.**047 Chi^2^**
CPT *z*-score	−0.89 (1.03) (−1.47, 0.20)	−0.98 (0.64) (−1.04, 0.18)	−0.87 (1.03) (−1.19, 0.28)	−0.98 (0.71) (−1.19, 0.58)	0.990 KW
CPT% abnormal	0%	0%	0%	0%	N/A
HPT *z*-score	−0.73 ± 1.01 (−2.02, 1.50)	−0.79 ± 0.98 (−1.92, 0.44)	−0.62 ± 1.62 (−2.03, 2.10)	−1.34 ± 0.52 (−2.03, −0.48)	0.253 A
HPT% abnormal	7.7%	0%	37.5%	23.5%	0.229 Chi^2^
PPT *z*-score	0.77 ± 0.93 (−0.55, 2.20)	1.31 ± 2.19 (−2.09, 3.86)	0.55 ± 1.89 (−1.92, 3.12)	1.19 ± 1.81 (−2.08, 4.41)	0.751 A
PPT% abnormal	7.7%	60%	25.0%	64.7%	**0**.**009 Chi^2^**
MPT *z*-score	1.24 ± 1.54 (−0.75, 4.41)	1.23 ± 1.57 (−0.54, 2.44)	−1.60 ± 2.11 (−3.14, 1.46)	−1.91 ± 2.02 (−3.24, 2.88)	**0**.**001 A**
MPT% abnormal	23.1%	60%	62.5%	82.4%	**0**.**013 Chi^2^**
MPS *z*-score	−0.08 (1.53) (−2.12, 2.48)	0.14 (2.53) (−1.10, 2.08)	−1.72 (1.56) (−2.12, 0.90)	−1.43 (1.31) (−2.12, 3.16)	**0**.**014 KW**
MPS% abnormal	38.5%	20.0%	75.0%	76.5%	**0**.**040 Chi^2^**
WUR *z*-score	−0.22 ± 1.03 (−1.33, 1.92)	−0.21 ± 1.49 (−2.25, 1.08)	−0.08 ± 0.86 (−1.14, 1.00)	−0.64 ± 0.95 (−1.72, 0.06)	0.919 A
WUR% abnormal	0%	20.0%	0%	0%	0.244 Chi^2^
MDT *z*-score	0.64 (1.97) (−2.05, 2.68)	0.26 (1.72) (−1.03, 2.05)	−1.31 (5.46) (−4.86, 2.19)	−1.74 (3.39) (−5.48, 0.64)	**0**.**002 KW**
MDT% abnormal	38.5%	20.0%	50.0%	47.1%	0.492 Chi^2^
VDT *z*-score	0.11 (1.53) (−2.87, 1.01)	−0.04 (2.16) (−2.96, 1.12)	−2.20 (3.83) (−5.53, 0.78)	−2.80 (1.94) (−5.53, 0.99)	**0**.**009 KW**
VDT% abnormal	15.4%	20.0%	62.5%	64.7%	**0**.**023 Chi^2^**
DMA% abnormal	0%	0%	0%	17.6%	0.177
PHS% abnormal	7.7%	60.0%	25.0%	47.1%	0.065 Chi^2^

The *z*-scores are provided as mean ± SD or median (IQR) and the range (minimum, maximum) for each group.

Boldface denotes *p* < 0.05.

Immunostaining for the pan-neuronal marker (PGP 9.5) in skin biopsies from the HV and no-DPN groups is shown in Figure 1. These were significantly reduced in DPN groups with pain or without pain (*p* < 0.0001 and *p* = 0.001, respectively). There was no statistical difference between HV vs. no-DPN groups and painful-DPN vs. painless DPN groups.

**Figure 2:**
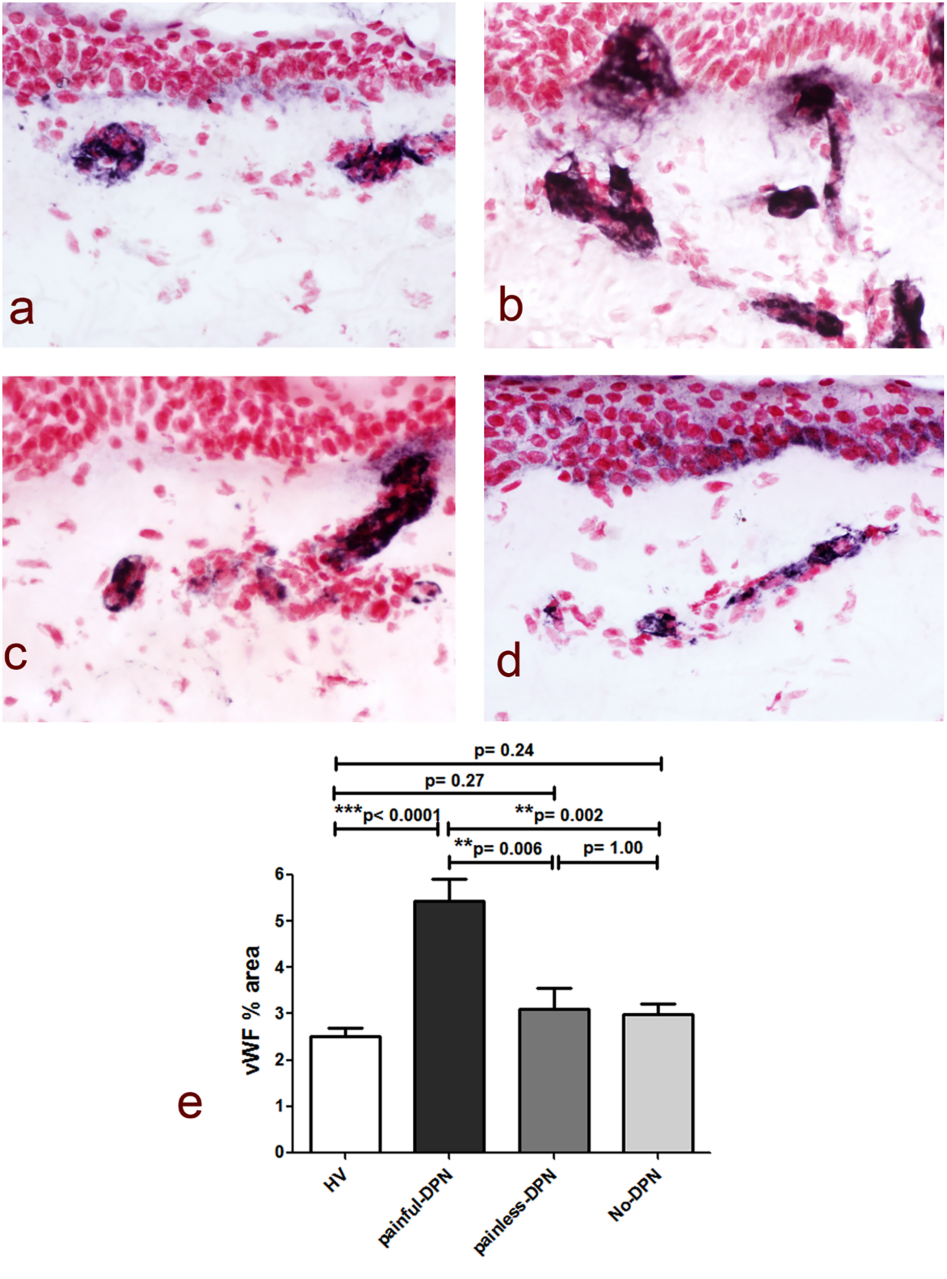
**(a)** vWF immunoreactivity in thigh skin from the healthy volunteers and the HV, **(b)** painful-DPN, **(c)** painless-DPN, and **(d)** no-DPN groups. Magnification ×40. **(e)** Image analysis of vWF subepidermal vessel immunostaining (% immunoreactivity).

Immunostaining for the vascular marker vWF showed a significant increase in the subepidermal staining area in the painful-DPN group (*p* < 0.0001) vs. the HV group (Figure 2). In contrast, there was no significant difference between the HV group and the painless-DPN group (*p* = 0.27), nor was this group different from the no-DPN group (*p* = 1.00). Importantly, the painful-DPN group was also significantly different from both the painless-DPN group and no-DPN group (*p* = 0.006 and *p* = 0.002, respectively).

**Figure 3:**
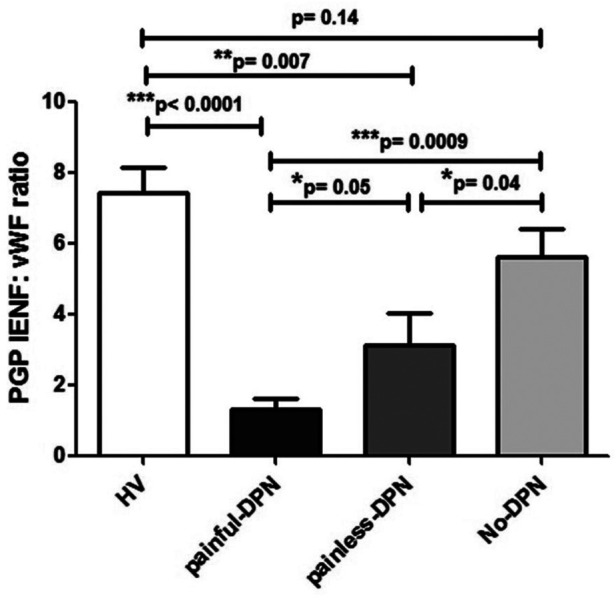
Bar charts showing image analyses (mean ± SEM) of PGP9.5 IENF: vWF ratios in thigh skin from the healthy volunteers and the HV, painful-DPN, painless-DPN, and no-DPN groups.

The ratio of PGP9.5 IENF to vWF showed a significant difference between painful- and painless-DPN groups (*p* = 0.05) (Figure 3).

Ten participants who had PGP 9.5 immunohistochemistry did not have vWF immunohistochemistry (HV: age 57.3 ± 12.2, female 57%, BMI 26.7 ± 6.3 kg/m^2^, and HbA1c 37.3 ± 5.1 mmol/mol; and painful-DSPN: age 67.7 ± 6.1, female 66.6%, BMI 28.9 ± 2.9 kg/m^2^, and HbA1c 67.0 ± 7.3 mmol/mol). There was an increase in vWF immunoreactivity in painful-DSPN [4.7 (0.9)% area] compared with all three other study groups vs. the HV [2.4 (1.0), Mann–Whitney *U*-test, *p* < 0.001], no-DSPN [(2.7 (1.1), *p* = 0.005], and painless-DSPN [3.1 (1.8), *p* = 0.001)] groups, as shown in Figure 2. Moreover, there was a significant group difference in the PGP9.5 IENF:vWF ratio as shown in Figure 3.

Table 4 demonstrates the correlation analysis between vWF immunoreactivity at the thigh and clinical, demographic, and neurological variables. vWF immunoreactivity positively correlated with clinical variables, including the waist/hip ratio and HbA1c. Additionally, vWF immunoreactivity also positively correlated with neuropathy measures including the clinical scoring systems (TCNS and NIS-LL), objective measures (nerve conduction studies and QST measures), and the composite score NIS-LL + 7 (Spearman's correlation, *r* = 0.641, *p* < 0.001). vWF also correlated with measures of neuropathic pain, including all the subscores of the NPSI and the total NPSI (*r* = 0.642, *p* < 0.001), DN4 (*r* = 0.659, *p* < 0.001), and VAS (*r* = 0.585, *p* = 0.004) scores. Finally, vWF also correlated with PGP 9.5 immunoreactivity (*r* = −0.571, *p* = 0.001).

**Table 4:** Spearman's correlation between vWF immunoreactivity in thigh skin and clinical, demographic, and neurological variables in study participants.

	*r*	*p*-value
Age	−0.154	0.392
Duration of DM	−0.120	0.552
ACR	0.318	0.071
Pack-years smoking	0.308	0.082
Waist/hip ratio	0.352	**0** **.** **044**
Body mass index	−0.032	0.859
Systolic blood pressure	0.212	0.245
Creatinine	0.115	0.526
Total cholesterol	−0.179	0.319
HbA1c	0.531	**0**.**001**
Burning spontaneous pain NPSI subscore	0.545	**0**.**001**
Pressing spontaneous pain NPSI subscore	0.536	**0**.**001**
Paroxysmal pain NPSI subscore	0.661	**<0**.**001**
Evoked pain NPSI subscore	0.590	**<0**.**001**
Paraesthesia/dysaesthesia NPSI subscore	0.482	**0**.**004**
NPSI	0.642	**<0**.**001**
DN4	0.659	**<0**.**001**
TCNS	0.752	**<0**.**001**
NIS-LL	0.713	**<0**.**001**
NIS-LL + 7	0.641	**<0**.**001**
Peroneal CMAP	−0.352	0.052
Peroneal MNCV	−0.154	0.444
Peroneal MNDL	0.182	0.344
Tibial MNDL	0.472	**0**.**007**
Sural SNAP	−0.576	0.001
Foot ESC	0.605	**<0**.**001**
VAS pain score	0.585	**0**.**004**
CDT *z*-score	−0.373	**0**.**033**
WDT *z*-score	−0.499	**0**.**003**
TSL *z*-score	−0.414	**0**.**017**
CPT *z*-score	−0.078	0.666
HPT *z*-score	−0.437	**0**.**011**
PPT *z*-score	0.148	0.410
MPT *z*-score	−0.488	**0**.**004**
MPS *z*-score	−0.221	0.217
WUR *z*-score	0.227	0.502
MDT *z*-score	−0.531	**0**.**001**
VDT *z*-score	−0.548	**0**.**001**
DMA *z*-score	N/A	N/A
PHS *z*-score	N/A	N/A
PGP 9.5 IENF	−0.571	**0**.**001**

Boldface denotes *p* < 0.05.

## Discussion

In this study, we report a significant increase in the vascular marker vWF in skin biopsies taken at the thigh in patients with painful DPN compared with all other study groups. Importantly, the increase in vWF was significantly greater in the painful- compared with the painless-DPN group, despite both groups having a similar severity of neuropathy. There was also a significant reduction in the PGP IENF:vWF ratio in the painful-DPN group compared with all other groups.

The findings of this study are comparable to those in our previous publication (24), although now confirmed at a different biopsy site and in a cohort with more detailed phenotyping. In this previous study, we performed immunohistochemical analysis of skin biopsies at the distal calf and found that IENF density was severely depleted in neuropathy groups but that vWF-positive blood vessels were significantly increased in the painful-DPN group. Thus, we performed skin biopsy at a more proximal site where IENF was less depleted. It is also notable that the vWF% area was numerically lower in thigh skin biopsies in painful DPN than at the calf, but the IENF density was higher in the thigh. This is expected in a length-dependent neuropathy, i.e., less advanced DPN at the thigh compared with the distal calf, and may also explain why pain symptoms present first in the feet and distal calf. We also demonstrated a correlation between clinical and neurophysiological measures and vWF immunoreactivity. Most notably, there were significant correlations with neuropathy severity but also measures of neuropathic pain, including NPSI total and subscores, VAS pain, and DN4. This therefore highlights the relationship between vWF and pain in DPN.

The mechanisms underlying the maintenance and generation of neuropathic pain in DPN remain unclear. This study and our previous publication indicate that skin vascular factors may be involved in the pathophysiology of the condition (24). It has previously been suggested that more severe neuropathy appears to be associated with painful DPN (22, 29, 30, 48); thus, individuals with painful DPN may undergo a more advanced hypoxia-induced increase in skin blood vessels, which then expose local small nociceptive fibers to a relative excess of algogens such as nerve growth factor (24, 49, 50). These surviving nociceptors will then be “over-trophed” and promote pain and sensitivity, as seen in NGF-induced inflammation in normal skin (49). Another study has also demonstrated evidence of hypoxia and abnormal angiogenesis in patients with painful DPN (51), who found hypoxia-inducible factor 1 α (HIF-1α), a measure of tissue hypoxia, immunostaining correlated with measures of pain in individuals with DPN. Moreover, in painful non-freezing cold injury (trench foot), a similar finding of increased vWF staining was also demonstrated (46).

In support of vascular factors being an important contributory factor to the generation and maintenance of neuropathic pain in DPN, various other studies have found alterations suggestive of vascular dysfunction in painful DPN (22). Several studies have shown peripheral blood flow regulation has been found to be altered in painful DPN compared with painless DPN (22, 52–55).

For example, we found elevated sural nerve epineural oxygen saturation and faster blood flow in patients with painful DPN compared to painless DPN, perhaps secondary to arteriovenous shunting (55). Moreover, studies measuring angiogenesis (vascular endothelial growth factor, VEGF) and endothelial dysfunction (soluble intercellular adhesion molecule 1, sICAM-1) have found them to be elevated in painful DPN (56, 57). Not only are peripheral vascular mechanisms thought to be involved in painful DPN but also central mechanisms. Thalamic and anterior cingulate cortical hypervascularity have been shown to be present in painful DPN (58, 59).

This study opens new lines of investigation. Firstly, although vWF has been used as a vascular marker in normal human tissues (60), vWF has also been found to be raised in conditions causing endothelial damage (61) and is also upregulated in angiogenesis (62, 63). Therefore, further study is necessary to determine the mechanistic link between vWF elevation and painful DPN and whether increased vWF staining is a result of a greater number of blood vessels, as it appears to be, or due to vascular alterations such as increased endothelial damage or greater angiogenesis. Secondly, this study does present a possible mechanistic link explaining the efficacy of topical vasodilatory agents in treating painful DPN (64, 65). In previous studies, we have demonstrated that clinical phenotyping using DFNS QST and neuroimaging markers can predict the treatment efficacy of intravenous lidocaine (42). Therefore, future studies could use skin vWF as a biomarker of painful DPN to determine whether it predicts the efficacy of topical vasodilatory treatment as a mechanistic treatment agent.

This study confirms that vascular density is increased in the skin in painful DPN and that dermal vasculature related to IENF density may lead to neuropathic pain, in accordance with our proposed mechanism. Future studies should investigate the molecular links between vascular density and painful DPN, thereby providing new targets for treatments of painful DPN.

## Data Availability

The original contributions presented in the study are included in the article/Supplementary Material, further inquiries can be directed to the corresponding author.

## References

[1] Sloan G Selvarajah D Tesfaye S. Pathogenesis, diagnosis and clinical management of diabetic sensorimotor peripheral neuropathy. Nat Rev Endocrinol. (2021) 17:400–20. 10.1038/s41574-021-00496-z 34050323

[2] Galer BS Gianas A Jensen MP. Painful diabetic polyneuropathy: epidemiology, pain description, and quality of life. Diabetes Res Clin Pract. (2000) 47:123–8. 10.1016/s0168-8227(99)00112-6 10670912

[3] Abbott CA Malik RA van Ross ERE Kulkarni J Boulton AJM. Prevalence and characteristics of painful diabetic neuropathy in a large community-based diabetic population in the U.K. Diabetes Care. (2011) 34:2220–4. 10.2337/dc11-1108 21852677 PMC3177727

[4] Tölle T Xu X Sadosky AB. Painful diabetic neuropathy: a cross-sectional survey of health state impairment and treatment patterns. J Diabetes Complicat. (2006) 20:26–33. 10.1016/j.jdiacomp.2005.09.007 16389164

[5] Selvarajah D Cash T Sankar A Thomas L Davies J Cachia E The contributors of emotional distress in painful diabetic neuropathy. Diab Vasc Dis Res. (2014) 11:218–25. 10.1177/1479164114522135 24821753

[6] Gore M Brandenburg NA Dukes E Hoffman DL Tai K-S Stacey B. Pain severity in diabetic peripheral neuropathy is associated with patient functioning, symptom levels of anxiety and depression, and sleep. J Pain Symptom Manage. (2005) 30:374–85. 10.1016/j.jpainsymman.2005.04.009 16256902

[7] Alleman CJM Westerhout KY Hensen M Chambers C Stoker M Long S Humanistic and economic burden of painful diabetic peripheral neuropathy in Europe: a review of the literature. Diabetes Res Clin Pract. (2015) 109:215–25. 10.1016/j.diabres.2015.04.031 26008721

[8] Feldman EL Nave K-A Jensen TS Bennett DLH. New horizons in diabetic neuropathy: mechanisms, bioenergetics, and pain. Neuron. (2017) 93:1296–313. 10.1016/j.neuron.2017.02.005 28334605 PMC5400015

[9] Anand P Bley K. Topical capsaicin for pain management: therapeutic potential and mechanisms of action of the new high-concentration capsaicin 8% patch. Br J Anaesth. (2011) 107:490–502. 10.1093/bja/aer260 21852280 PMC3169333

[10] Anand P Privitera R Donatien P Fadavi H Tesfaye S Bravis V Reversing painful and non-painful diabetic neuropathy with the capsaicin 8% patch: clinical evidence for pain relief and restoration of function via nerve fiber regeneration. Front Neurol. (2022) 13:998904. 10.3389/fneur.2022.998904 36388188 PMC9643187

[11] Anand P Privitera R Donatien P Misra VP Woods DR. Capsaicin 8% patch treatment in non-freezing cold injury: evidence for pain relief and nerve regeneration. Front Neurol. (2021) 12:722875. 10.3389/fneur.2021.722875 34489857 PMC8418325

[12] Privitera R Anand P. Capsaicin 8% patch Qutenza and other current treatments for neuropathic pain in chemotherapy-induced peripheral neuropathy (CIPN). Curr Opin Support Palliat Care. (2021) 15:125–31. 10.1097/SPC.0000000000000545 33905384

[13] Freynhagen R Argoff C Eerdekens M Engelen S Perrot S. Progressive response to repeat application of capsaicin 179 mg (8% w/w) cutaneous patch in peripheral neuropathic pain: comprehensive new analysis and clinical implications. Pain Med. (2021) 22:2324–36. 10.1093/pm/pnab113 33871648 PMC8500721

[14] Martini C Yassen A Olofsen E Passier P Stoker M Dahan A. Pharmacodynamic analysis of the analgesic effect of capsaicin 8% patch (Qutenza™) in diabetic neuropathic pain patients: detection of distinct response groups. J Pain Res. (2012) 5:51–9. 10.2147/JPR.S30406 22536092 PMC3333798

[15] Vinik AI Perrot S Vinik EJ Pazdera L Jacobs H Stoker M Capsaicin 8% patch repeat treatment plus standard of care (SOC) versus SOC alone in painful diabetic peripheral neuropathy: a randomised, 52-week, open-label, safety study. BMC Neurol. (2016) 16:251. 10.1186/s12883-016-0752-7 27919222 PMC5139122

[16] Sloan G Alam U Selvarajah D Tesfaye S. The treatment of painful diabetic neuropathy. Curr Diabetes Rev. (2022) 18:e070721194556. 10.2174/1573399817666210707112413 34238163

[17] Tesfaye S Sloan G Petrie J White D Bradburn M Julious S Comparison of amitriptyline supplemented with pregabalin, pregabalin supplemented with amitriptyline, and duloxetine supplemented with pregabalin for the treatment of diabetic peripheral neuropathic pain (OPTION-DM): a multicentre, double-blind, randomised crossover trial. Lancet. (2022) 400:680–90. 10.1016/S0140-6736(22)01472-6 36007534 PMC9418415

[18] Cheung A Podgorny P Martinez JA Chan C Toth C. Epidermal axonal swellings in painful and painless diabetic peripheral neuropathy. Muscle Nerve. (2015) 51:505–13. 10.1002/mus.24351 25130671

[19] Scheytt S Riediger N Braunsdorf S Sommer C Üçeyler N. Increased gene expression of growth associated protein-43 in skin of patients with early-stage peripheral neuropathies. J Neurol Sci. (2015) 355:131–7. 10.1016/j.jns.2015.05.044 26071889

[20] Bönhof GJ Strom A Püttgen S Ringel B Brüggemann J Bódis K Patterns of cutaneous nerve fibre loss and regeneration in type 2 diabetes with painful and painless polyneuropathy. Diabetologia. (2017) 60:2495–503. 10.1007/s00125-017-4438-5 28914336

[21] Galosi E La Cesa S Di Stefano G Karlsson P Fasolino A Leone C A pain in the skin. Regenerating nerve sprouts are distinctly associated with ongoing burning pain in patients with diabetes. Eur J Pain. (2018) 22:1727–34. 10.1002/ejp.1259 29885017

[22] Shillo P Sloan G Greig M Hunt L Selvarajah D Elliott J Painful and painless diabetic neuropathies: what is the difference? Curr Diab Rep. (2019) 19:32. 10.1007/s11892-019-1150-5 31065863 PMC6505492

[23] Karlsson P Provitera V Caporaso G Stancanelli A Saltalamacchia AM Borreca I Increased peptidergic fibers as a potential cutaneous marker of pain in diabetic small fiber neuropathy. Pain. (2021) 162:778–86. 10.1097/j.pain.0000000000002054 32833793

[24] Shillo P Yiangou Y Donatien P Greig M Selvarajah D Wilkinson ID Nerve and vascular biomarkers in skin biopsies differentiate painful from painless peripheral neuropathy in type 2 diabetes. Front Pain Res. (2021) 2:731658. 10.3389/fpain.2021.731658 PMC8915761 35295465

[25] Hoeijmakers JG Faber CG Lauria G Merkies IS Waxman SG. Small-fibre neuropathies–advances in diagnosis, pathophysiology and management. Nat Rev Neurol. (2012) 8:369–79. 10.1038/nrneurol.2012.97 22641108

[26] Tesfaye S Boulton AJM Dyck PJ Freeman R Horowitz M Kempler P Diabetic neuropathies: update on definitions, diagnostic criteria, estimation of severity, and treatments. Diabetes Care. (2010) 33:2285–93. 10.2337/dc10-1303 20876709 PMC2945176

[27] Pop-Busui R Boulton AJM Feldman EL Bril V Freeman R Malik RA Diabetic neuropathy: a position statement by the American Diabetes Association. Diabetes Care. (2017) 40:136–54. 10.2337/dc16-2042 27999003 PMC6977405

[28] Kennedy WR Wendelschafer-Crabb G Johnson T. Quantitation of epidermal nerves in diabetic neuropathy. Neurology. (1996) 47:1042–8. 10.1212/wnl.47.4.1042 8857742

[29] Themistocleous AC Ramirez JD Shillo PR Lees JG Selvarajah D Orengo C The Pain in Neuropathy Study (PiNS): a cross-sectional observational study determining the somatosensory phenotype of painful and painless diabetic neuropathy. Pain. (2016) 157:1132–45. 10.1097/j.pain.0000000000000491 27088890 PMC4834814

[30] Raputova J Srotova I Vlckova E Sommer C Üçeyler N Birklein F Sensory phenotype and risk factors for painful diabetic neuropathy: a cross-sectional observational study. Pain. (2017) 158:2340–53. 10.1097/j.pain.0000000000001034 28858986 PMC5690294

[31] Sloan G Shillo P Selvarajah D Wu J Wilkinson ID Tracey I A new look at painful diabetic neuropathy. Diabetes Res Clin Pract. (2018) 144:177–91. 10.1016/j.diabres.2018.08.020 30201394

[32] Cheng HT Dauch JR Porzio MT Yanik BM Hsieh W Smith AG Increased axonal regeneration and swellings in intraepidermal nerve fibers characterize painful phenotypes of diabetic neuropathy. J Pain. (2013) 14:941–7. 10.1016/j.jpain.2013.03.005 23685187 PMC3994562

[33] Lauria G Hsieh ST Johansson O Kennedy WR Leger JM Mellgren SI European Federation of Neurological Societies/Peripheral Nerve Society guideline on the use of skin biopsy in the diagnosis of small fiber neuropathy. Report of a joint task force of the European Federation of Neurological Societies and the Peripheral Nerve Society. Eur J Neurol. (2010) 17:903–12; e44–9. 10.1111/j.1468-1331.2010.03023.x 20642627

[34] Bril V Perkins BA. Validation of the Toronto Clinical Scoring System for diabetic polyneuropathy. Diabetes Care. (2002) 25:2048–52. 10.2337/diacare.25.11.2048 12401755

[35] Dyck PJ Litchy WJ Daube JR Harper CM Dyck PJB Davies J Individual attributes versus composite scores of nerve conduction abnormality: sensitivity, reproducibility, and concordance with impairment. Muscle Nerve. (2003) 27:202–10. 10.1002/mus.10320 12548528

[36] Bouhassira D Attal N Alchaar H Boureau F Brochet B Bruxelle J Comparison of pain syndromes associated with nervous or somatic lesions and development of a new neuropathic pain diagnostic questionnaire (DN4). Pain. (2005) 114:29–36. 10.1016/j.pain.2004.12.010 15733628

[37] Bouhassira D Attal N Fermanian J Alchaar H Gautron M Masquelier E Development and validation of the neuropathic pain symptom inventory. Pain. (2004) 108:248–57. 10.1016/j.pain.2003.12.024 15030944

[38] Casellini CM Parson HK Richardson MS Nevoret ML Vinik AI. Sudoscan, a noninvasive tool for detecting diabetic small fiber neuropathy and autonomic dysfunction. Diabetes Technol Ther. (2013) 15:948–53. 10.1089/dia.2013.0129 23889506 PMC3817891

[39] Dyck PJ O’Brien PC Kosanke JL Gillen DA Karnes JL. A 4, 2, and 1 stepping algorithm for quick and accurate estimation of cutaneous sensation threshold. Neurology. (1993) 43:1508–12. 10.1212/wnl.43.8.1508 8351003

[40] O’Brien IA O’Hare P Corrall RJ. Heart rate variability in healthy subjects: effect of age and the derivation of normal ranges for tests of autonomic function. Br Heart J. (1986) 55:348–54. 10.1136/hrt.55.4.348 3964501 PMC1236737

[41] Rolke R Baron R Maier C Tölle TR Treede DR Beyer A Quantitative sensory testing in the German research network on neuropathic pain (DFNS): standardized protocol and reference values. Pain. (2006) 123:231–43. 10.1016/j.pain.2006.01.041 16697110

[42] Teh K Wilkinson ID Heiberg-Gibbons F Awadh M Kelsall A Pallai S Somatosensory network functional connectivity differentiates clinical pain phenotypes in diabetic neuropathy. Diabetologia. (2021) 64:1412–21. 10.1007/s00125-021-05416-4 33768284 PMC8099810

[43] Magerl W Krumova EK Baron R Tölle T Treede R-D Maier C. Reference data for quantitative sensory testing (QST): refined stratification for age and a novel method for statistical comparison of group data. Pain. (2010) 151:598–605. 10.1016/j.pain.2010.07.026 20965658

[44] Dyck PJ Davies JL Litchy WJ O’Brien PC. Longitudinal assessment of diabetic polyneuropathy using a composite score in the Rochester Diabetic Neuropathy Study cohort. Neurology. (1997) 49:229–39. 10.1212/wnl.49.1.229 9222195

[45] Jensen TS Baron R Haanpää M Kalso E Loeser JD Rice ASC A new definition of neuropathic pain. Pain. (2011) 152:2204–5. 10.1016/j.pain.2011.06.017 21764514

[46] Anand P Privitera R Yiangou Y Donatien P Birch R Misra P. Trench foot or non-freezing cold injury as a painful vaso-neuropathy: clinical and skin biopsy assessments. Front Neurol. (2017) 8:514. 10.3389/fneur.2017.00514 28993756 PMC5626869

[47] Facer P Casula MA Smith GD Benham CD Chessell IP Bountra C Differential expression of the capsaicin receptor TRPV1 and related novel receptors TRPV3, TRPV4 and TRPM8 in normal human tissues and changes in traumatic and diabetic neuropathy. BMC Neurol. (2007) 7:11. 10.1186/1471-2377-7-11 17521436 PMC1892784

[48] Hébert HL Veluchamy A Torrance N Smith BH. Risk factors for neuropathic pain in diabetes mellitus. Pain. (2017) 158:560–8. 10.1097/j.pain.0000000000000785 27941499 PMC5359789

[49] Anand P. Neurotrophins and peripheral neuropathy. Philos Trans R Soc Lond B Biol Sci. (1996) 351:449–54. 10.1098/rstb.1996.0041 8730784

[50] Anand P Terenghi G Warner G Kopelman P Williams-Chestnut RE Sinicropi DV. The role of endogenous nerve growth factor in human diabetic neuropathy. Nat Med. (1996) 2:703–7. 10.1038/nm0696-703 8640566

[51] Quattrini C Jeziorska M Boulton AJM Malik RA. Reduced vascular endothelial growth factor expression and intra-epidermal nerve fiber loss in human diabetic neuropathy. Diabetes Care. (2008) 31:140–5. 10.2337/dc07-1556 17934147

[52] Archer AG Roberts VC Watkins PJ. Blood flow patterns in painful diabetic neuropathy. Diabetologia. (1984) 27:563–7. 10.1007/BF00276968 6530051

[53] Tsigos C White A Young RJ. Discrimination between painful and painless diabetic neuropathy based on testing of large somatic nerve and sympathetic nerve function. Diabet Med. (1992) 9:359–65. 10.1111/j.1464-5491.1992.tb01797.x 1600708

[54] Tack CJ van Gurp PJ Holmes C Goldstein DS. Local sympathetic denervation in painful diabetic neuropathy. Diabetes. (2002) 51:3545–53. 10.2337/diabetes.51.12.3545 12453912

[55] Eaton SEM Harris ND Ibrahim S Patel KA Selmi F Radatz M Increased sural nerve epineurial blood flow in human subjects with painful diabetic neuropathy. Diabetologia. (2003) 46:934–9. 10.1007/s00125-003-1127-3 12819899

[56] Doupis J Lyons TE Wu S Gnardellis C Dinh T Veves A. Microvascular reactivity and inflammatory cytokines in painful and painless peripheral diabetic neuropathy. J Clin Endocrinol Metab. (2009) 94:2157–63. 10.1210/jc.2008-2385 19276232 PMC2690431

[57] Herder C Bongaerts BWC Rathmann W Heier M Kowall B Koenig W Differential association between biomarkers of subclinical inflammation and painful polyneuropathy: results from the KORA F4 study. Diabetes Care. (2015) 38:91–6. 10.2337/dc14-1403 25325880

[58] Watanabe K Hirano S Kojima K Nagashima K Mukai H Sato T Altered cerebral blood flow in the anterior cingulate cortex is associated with neuropathic pain. J Neurol Neurosurg Psychiatr. (2018) 89:1082–7. 10.1136/jnnp-2017-316601 29627772

[59] Selvarajah D Wilkinson ID Gandhi R Griffiths PD Tesfaye S. Microvascular perfusion abnormalities of the thalamus in painful but not painless diabetic polyneuropathy: a clue to the pathogenesis of pain in type 1 diabetes. Diabetes Care. (2011) 34:718–20. 10.2337/dc10-1550 21282344 PMC3041213

[60] Pusztaszeri MP Seelentag W Bosman FT. Immunohistochemical expression of endothelial markers CD31, CD34, von Willebrand factor, and Fli-1 in normal human tissues. J Histochem Cytochem. (2006) 54:385–95. 10.1369/jhc.4A6514.2005 16234507

[61] Mannucci PM. von Willebrand factor: a marker of endothelial damage? Arterioscler Thromb Vasc Biol. (1998) 18:1359–62. 10.1161/01.atv.18.9.1359 9743222

[62] Zanetta L Marcus SG Vasile J Dobryansky M Cohen H Eng K Expression of von Willebrand factor, an endothelial cell marker, is up-regulated by angiogenesis factors: a potential method for objective assessment of tumor angiogenesis. Int J Cancer. (2000) 85:281–8. 10.1002/(sici)1097-0215(20000115)85:2 &lt; 281::aid-ijc21>3.0.co;2-3 10629090 10.1002/(sici)1097-0215(20000115)85:2<281::aid-ijc21>3.0.co;2-3

[63] Randi AM Laffan MA. Von Willebrand factor and angiogenesis: basic and applied issues. J Thromb Haemost. (2017) 15:13–20. 10.1111/jth.13551 27778439

[64] Agrawal RP Choudhary R Sharma P Sharma S Beniwal R Kaswan K Glyceryl trinitrate spray in the management of painful diabetic neuropathy: a randomized double blind placebo controlled cross-over study. Diabetes Res Clin Pract. (2007) 77:161–7. 10.1016/j.diabres.2006.12.003 17316865

[65] Agrawal RP Goswami J Jain S Kochar DK. Management of diabetic neuropathy by sodium valproate and glyceryl trinitrate spray: a prospective double-blind randomized placebo-controlled study. Diabetes Res Clin Pract. (2009) 83:371–8. 10.1016/j.diabres.2008.12.018 19208440

